# Robot-Assisted Laparoscopic Nephroureterectomy for Transitional Cell Carcinoma of a Right Pelvic Kidney

**DOI:** 10.1089/cren.2016.0068

**Published:** 2016-07-01

**Authors:** Michael E. Rezaee, Zubin Shetty, David Pridmore, Chirag N. Dave, Sugandh D. Shetty

**Affiliations:** ^1^ Oakland University William Beaumont School of Medicine, Rochester, Michigan.; ^2^ Wayne State University School of Medicine, Detroit, Michigan.; ^3^ Department of Urology, William Beaumont Hospital, Beaumont Health, Royal Oak, Michigan.

## Abstract

***Background:*** Nephroureterectomy is the standard of care for transitional cell carcinoma (TCC) involving the upper urinary tract. However, few published case reports exist describing the surgical treatment of ectopic kidneys with TCC. Surgical removal of a pelvic kidney can be complicated by aberrant vasculature supply, a tortuous ureter and abutting anatomical structures. Thus, it is necessary to determine the most appropriate surgical technique for treatment of pelvic kidneys with suspected malignancy.

***Case Presentation:*** A 65-year-old female who presented with hematuria and lower abdominal pain was found to have a right pelvic kidney with a heterogeneous mass on computed tomography (CT) urogram. A robot-assisted laparoscopic nephroureterectomy of the right pelvic kidney was performed. Histopathological analysis revealed high-grade TCC with microscopic extension through the muscularis propria of the renal pelvis and superficially into the renal parenchyma.

***Conclusion:*** This case demonstrates the successful use of robot-assisted laparoscopic nephroureterectomy in the treatment of a pelvic kidney with TCC. Preoperative CT angiography is critical to define vascular anatomy and to prevent significant blood loss and damage to surrounding structures during surgery. This case was presented because TCC of a pelvic kidney is a rare occurrence and the use of robot-assisted nephroureterectomy for treatment of this disease is novel.

## Background

The occurrence of a pelvic kidney is relatively uncommon. It has been estimated that 1 in every 1000 adults carries this congenital anomaly.^1^ Although ectopic kidneys are associated with increased risk for nephrolithiasis, hydronephrosis, and infection, they are not thought to be subject to increased rates of malignancy. In 2015, kidney cancer was found to be the ninth most common type of newly diagnosed cancer in the U.S., accounting for 3.7% of all cancer cases in the country.^2^ Transitional cell carcinoma (TCC) of the renal pelvis accounted for ∼10% of these cancers.^2^ The prevalence of TCC within a pelvic kidney is unknown, but is exceedingly rare. Nephroureterectomy is the standard of care for localized upper tract TCC. However, few published case reports exist describing the surgical treatment of ectopic kidneys with TCC. Surgical removal of a pelvic kidney can be complicated by aberrant vasculature supply, a tortuous ureter, and abutting anatomical structures. Thus, it is necessary to determine the most appropriate surgical technique for the treatment of pelvic kidneys with suspected malignancy.

In this study, we present the case of a 65-year-old female who presented with hematuria and lower abdominal pain. She was found to have a right pelvic kidney containing a heterogeneous mass on computed tomography (CT) urogram. A successful robot-assisted laparoscopic nephroureterectomy of the right pelvic kidney was performed. Histopathological analysis revealed high-grade TCC with microscopic extension through the muscularis propria of the renal pelvis and into the renal parenchyma.

## Case Presentation

A 65-year-old female presented with complaints of gross hematuria and lower abdominal pain. She denied flank pain, dysuria, urinary urgency or frequency, unexplained weight loss, or fatigue. Past medical history included cervical intraepithelial neoplasia III and endometrial hyperplasia, for which she received a fractional dilatation and curettage and complete conization. Although she had quit a decade before presentation, the patient had a 70.5 pack-year smoking history. Physical examination was unremarkable. A complete metabolic panel and blood count with differential were normal. Repeat Pap smear and vaginosis screen were negative. Cancer antigen 125 was normal at 13 U/mL. Urine analysis with microscopic examination revealed 3+ red blood cells, 2+ protein, normal white blood cells, and no bacteria. Urine cytology was suspicious for malignancy of the urothelium. Fluorescence *in situ* hybridization testing was negative.

**FIG. 1.**
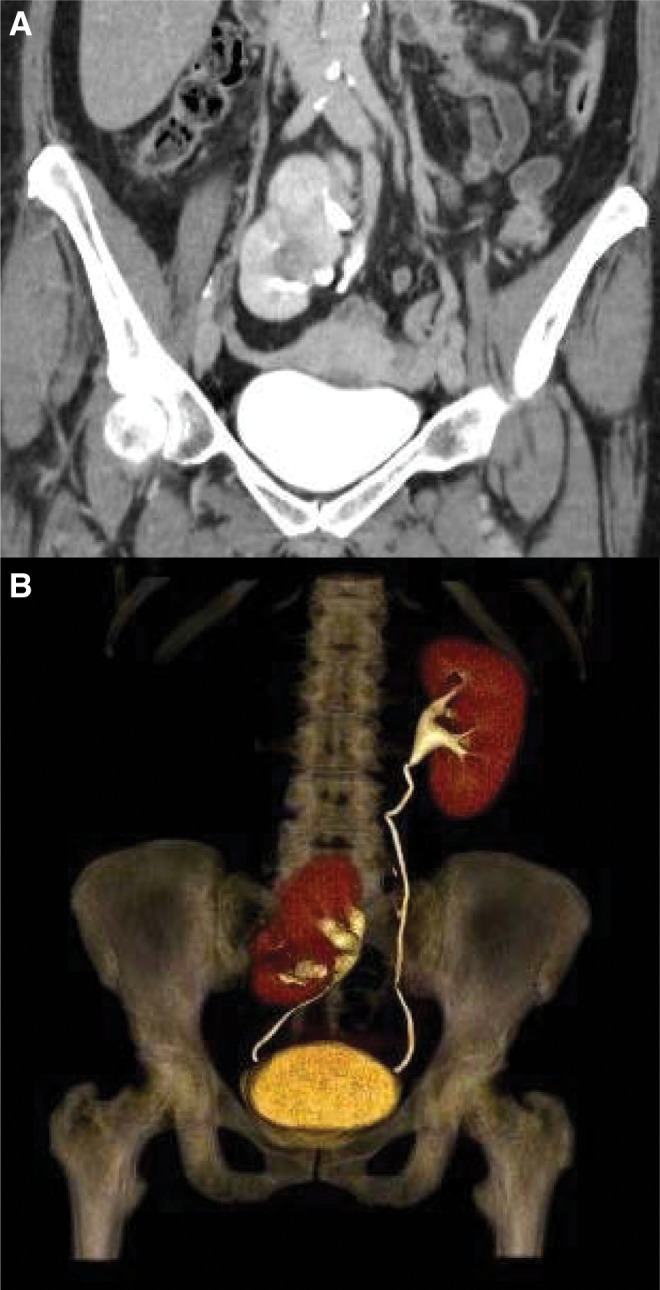
**(A)** Computed tomography (CT) urogram demonstrating a 3.6 × 2.0 cm heterogeneous mass originating from a right pelvic kidney, **(B)** CT urogram with three-dimensional (3D) reconstruction demonstrating a right pelvic kidney with associated dilation and distortion of the proximal renal collecting system.

**FIG. 2.**
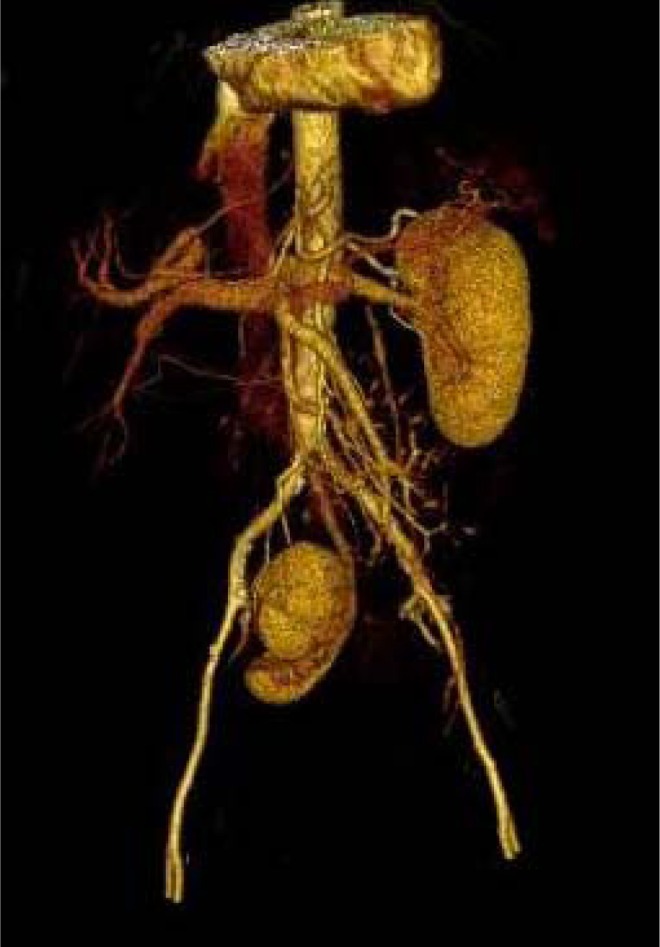
CT angiography of the abdomen and pelvis with 3D reconstruction displaying a single right renal artery originating from proximal right common iliac artery.

We obtained a CT urogram with three-dimensional (3D) reconstruction, which demonstrated a 3.6 × 2.0 cm heterogeneous mass originating from the right pelvic kidney with associated dilation of the proximal renal collecting system (Fig. 1). Imaging could not discern whether the mass originated from the renal parenchyma or the urothelium. Cystourethroscopy showed a normal-appearing urethra, bladder, and ureteral orifices. Retrograde pyelogram revealed a normal ureter and pelvicaliceal system on the left, but a pelvic kidney with a single, normal ureter and mildly dilated pelvicaliceal system with poor filling of the renal pelvis on the right. The hilum of the pelvic kidney was facing left. To examine vascular supply, a CT angiogram was obtained (Fig. 2). A single renal artery originated from the proximal right common iliac artery just distal to the aortic bifurcation, whereas a single renal vein emptied into the left common iliac vein bifurcation (Fig. 1). Biopsy of the mass was not attempted before surgery.

**FIG. 3.**
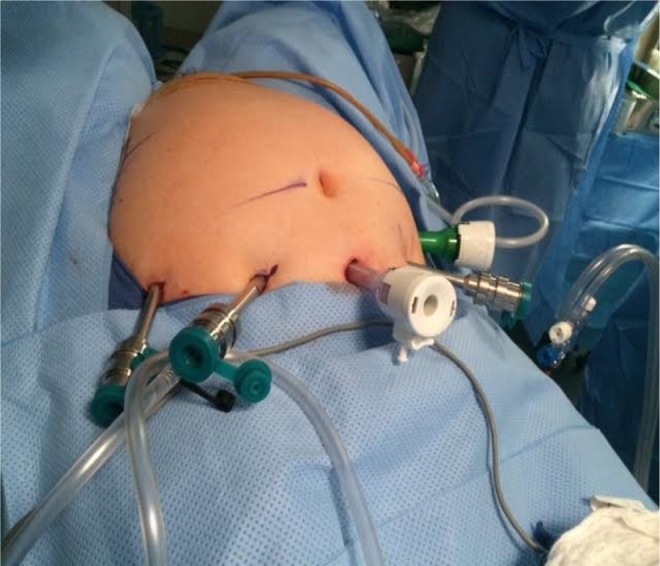
Port placement for right pelvic kidney nephroureterectomy.

A robot-assisted laparoscopic nephroureterectomy was performed based on the clinical workup strongly suggesting malignancy of the urothelium. The pelvic kidney was at the level of the sacral promontory, which required robotic port placement slightly cranial to the standard pelvic surgery. Therefore, a 12-mm ENDOPATH (Ethicon, Somerville, NJ) port was placed four fingerbreadths above the umbilicus. An 8-mm robotic port was placed five fingerbreadths lateral to the camera on the right side above the line of the umbilicus. A 15-mm port was placed in the right iliac fossa, whereas an 8-mm was placed in the left. An 8-mm left robotic port was placed four fingerbreadths lateral to the camera port cranial to the umbilical line (Fig. 3).

The ectopic kidney was found hanging by the aortic bifurcation. The peritoneum was incised along the right external iliac artery and traced proximally. Dissection was carried proximally around the bifurcation and then down the left common iliac vessels. A single renal artery originating from the proximal right common iliac artery was identified. A large renal vein draining into the left common iliac vein was found and prompted further dissection for better identification. A second smaller renal vein was found emptying into the inferior vena cava. A sufficient segment of the right renal artery was freed and then stapled with a 45 mm load. The main renal vein emptying to the left common iliac vein was then stapled in a similar manner. The right-sided accessory vein was then clipped. The kidney was lifted off the iliac vessels and attention was shifted to the ureter.

The ureter was traced to the ureterovesical junction and a cuff of the urinary bladder was excised. The opening in the bladder was sutured using 2-0 V-Loc (Covidien, New Haven, CT) and a hemostatic agent was placed after removing the specimen. Estimated blood loss was 20 mL. There were no postoperative complications.

The final pathology revealed a 3.8 cm tumor of the renal pelvis that was a high-grade, invasive papillary urothelial carcinoma with focal glandular differentiation (tubules, signet right cells, and mucin). The tumor extended through the muscularis propria of the renal pelvis, invading into the renal parenchyma. The patient subsequently underwent three cycles of adjuvant chemotherapy with methotrexate, vinblastine, doxorubicin, and cisplatin. The patient remains healthy without reoccurrence 18 months after her nephroureterectomy.

## Discussion and Literature Review

The prevalence of renal cancer in pelvic kidneys is exceedingly rare. Even more unique is the presence of TCC within a pelvic kidney. TCC accounts for less than 10% of all renal cancers diagnosed in the U.S. each year.^2^ Nephroureterectomy has been well established as the standard of care for this disease. The utility of robot-assisted laparoscopic nephroureterectomy of a pelvic kidney with TCC is largely unknown. We are unaware of any published cases of nephroureterectomy for TCC within a pelvic kidney at this time. To date, only a few case reports exist that describe the management of pelvic kidneys with suspected malignancy. Final pathology for most of these cases revealed renal cell carcinoma.

Two case reports have discussed the feasibility of robot-assisted partial nephrectomy for the treatment of suspected malignancy in a pelvic kidney.^3,4^ Both cases achieved successful resections with adequate margins and no postoperative complications. These reports describe how the use of the robot was advantageous in terms of visualization, operating in the pelvis without complication, and identifying and navigating the complex vasculature supply of and surrounding the pelvic kidney.

Unlike these cases, ours was a case of suspected TCC of the renal pelvis. We could not discern whether the mass originated from the renal parenchyma or urothelium, although urine cytology was suspicious. Nephroureterectomy provided the best chance of treatment. Of note, we did not attempt to biopsy the mass before surgery. Biopsy is often conducted in orthotopic kidneys with suspected malignancy. However, the risk of complications, such as perforation in the peritoneal cavity, may be elevated with pelvic kidneys. Therefore, we did not risk such a procedure. In addition, CT angiography to help identify aberrant vasculature was strongly suggested by previous case report authors.^3^ One pelvic kidney containing papillary renal cell carcinoma was found to have 5 renal arteries.^4^

We conducted a successful laparoscopic-assisted nephroureterectomy of a pelvic kidney with TCC. The robot provided optimal visualization of the kidney and was ergonomically advantageous for working in the pelvis. A CT urogram with 3D reconstruction was helpful in planning our surgical approach. A CT angiogram allowed us to examine the renal vasculature before surgery. For robotic pelvic kidney cases, special attention should be placed on the location of the ectopic kidney in relation to major vascular structures, the number and path of renal vessels, and the course of the ureter. Despite comprehensive imaging, aberrant vessels and their course may not be fully appreciated until the kidney is visualized intraoperatively.

## Conclusion

This case demonstrates the successful use of robot-assisted laparoscopic nephroureterectomy in the treatment of a pelvic kidney with TCC. Preoperative CT angiography is critical to define vascular anatomy and prevent significant blood loss and damage to surrounding structures during robotic pelvic kidney surgery. This case was presented because TCC of a pelvic kidney is a rare occurrence and the use of robot-assisted nephroureterectomy for treatment of this disease is a novel approach.

## Abbreviations Used

CT: computed tomography
TCC: transitional cell carcinoma
